# Epidemiological links and antimicrobial resistance of clinical *Salmonella enterica* ST198 isolates: a nationwide microbial population genomic study in Switzerland

**DOI:** 10.1099/mgen.0.000877

**Published:** 2022-10-27

**Authors:** Michael Biggel, Jule Horlbog, Magdalena Nüesch-Inderbinen, Marie Anne Chattaway, Roger Stephan

**Affiliations:** ^1^​ Institute for Food Safety and Hygiene, Vetsuisse Faculty University of Zürich, Zürich, Switzerland; ^2^​ National Reference Center for Enteropathogenic Bacteria and Listeria (NENT), Institute for Food Safety and Hygiene, Vetsuisse Faculty, University of Zurich, 8057 Zurich, Switzerland; ^3^​ Gastrointestinal Bacteria Reference Unit, UK Health Security Agency, London, UK

**Keywords:** ciprofloxacin resistance, genomics, population structure, prolonged *Salmonella* carriage, *ramR*, *Salmonella enterica* Kentucky ST198

## Abstract

*
Salmonella* is a leading cause of foodborne outbreaks and systemic infections worldwide. Emerging multi-drug resistant *Salmonella* lineages such as a ciprofloxacin-resistant subclade (CIP^R^) within *Salmonella enterica* serovar Kentucky ST198 threaten the effective prevention and treatment of infections. To understand the genomic diversity and antimicrobial resistance gene content associated with S. Kentucky in Switzerland, we whole-genome sequenced 70 human clinical isolates obtained between 2010 and 2020. Most isolates belonged to ST198-CIP^R^. High- and low-level ciprofloxacin resistance among CIP^R^ isolates was associated with variable mutations in *ramR* and *acrB* in combination with stable mutations in quinolone-resistance determining regions (QRDRs). Analysis of isolates from patients with prolonged ST198 colonization indicated subclonal adaptions with the *ramR* locus as a mutational hotspot. SNP analyses identified multiple clusters of near-identical isolates, which were often associated with travel but included spatiotemporally linked isolates from Switzerland. The largest SNP cluster was associated with travellers returning from Indonesia, and investigation of global data linked >60 additional ST198 salmonellosis isolates to this cluster. Our results emphasize the urgent need for implementing whole-genome sequencing as a routine tool for *Salmonella* surveillance and outbreak detection.

## Data Summary

Sequencing read data and genome assemblies of *S. enterica* isolates have been deposited at NCBI under BioProject accession number PRJNA803326. Individual accession numbers of newly sequenced Swiss isolates and associated metadata are listed in (Table S1, available in the online version of this article). Accession numbers of previously sequenced genomes from global isolates are listed in Table S6.

**Impact Statement**

The multi-drug resistant S. Kentucky ST198 CIP^R^ subclade has first emerged in the 1990s in Africa and subsequently spread to Asia and Europe. Our data confirm a predominance of the CIP^R^ subclade among human infections in Switzerland. Cases were however often travel-associated, with multiple infections caused by a clonal strain linked to Indonesia. Prolonged carriage of ST198 was relatively common and persisting strains often contained inactivating mutations in *ramR*, leading to high-level ciprofloxacin resistance. Our study provides genomic insights into the population structure, resistome, and evolution of this pathogen.

## Introduction

*Salmonella enterica* (* S. enterica *) is a major cause of foodborne disease in humans. In the European Union, *Salmonella* causes up to 100 000 gastrointestinal infections annually, making it the most common zoonotic pathogen after *Campylobacter* [1]. *Salmonellosis* typically presents with non-severe symptoms and is self-limited. Treatment with antibiotics (commonly ciprofloxacin, third-generation cephalosporins, or azithromycin) is however critical for patients with persistent or severe disease, which particularly affects immunocompromised or debilitated patients [2]. The emergence and spread of antimicrobial resistance (AMR) in *Salmonella* is thus a serious concern for public health. Ciprofloxacin-resistant *Salmonella* are classified as high-priority pathogens on the WHO list of resistant bacteria for which research and development strategies are urgently needed [3]. Ciprofloxacin resistance or reduced susceptibility has emerged in various *S. enterica* serotypes due to target mutations in quinolone-resistance determining regions (QRDRs) of DNA gyrase (*gyrA* and *gyrB*) and type IV topoisomerase (*parC* and *parE*) or the acquisition of plasmid-mediated quinolone resistance genes (PMQR; including *aac(6′)lb-cr*, *oqxAB*, *qepA*, *qnrA*, *qnrB*, *qnrC*, *qnrD*, and *qnrS*) [4–8]. Mutations affecting the functionality or expression of intrinsic porins such as OmpF or multi-drug efflux pumps such as the AcrAB-TolC complex also affect fluoroquinolone susceptibility [9, 10].

One of the *S. enterica* serovars notorious for its high rate of antimicrobial resistance is *S. enterica* subspecies *enterica* serovar Kentucky (*S*. Kentucky). The *S*. Kentucky serovar distribution is polyphyletic and encompasses distantly related lineages including ST314, ST152, and ST198. The latter comprises two major clades, ST198.1 and ST198.2 [11, 12]. Multi-drug resistant *S*. Kentucky isolates typically belong to a specific subclade (ST198-CIP^R^) embedded within ST198.2. ST198-CIP^R^ is characterized by chromosomal mutations in QRDRs conferring resistance against nalidixic acid and ciprofloxacin, namely mutations in *gyrA* codon 83 (S83F) and *parC* codon 80 (S80I), as well as one of three possible mutations in *gyrA* codon 87 (D87G, D87N, or D87Y), which are required for full ciprofloxacin resistance [13]. Subclade ST198-CIP^R^ isolates are frequently resistant to additional antibiotics including aminoglycosides, beta-lactams, sulfamethoxazole, and tetracycline [13, 14]. The acquisition of the resistance genes *aac(3)-Id* (also termed *aacA5*), *aadA7*, *bla*_TEM-1_, *sul1*, and *tetA* was linked to the chromosomal integration of the transferable *Salmonella* genomic island (SGI) 1 variant SGI1-K [13].

Phylogenomic analyses suggested that ST198-CIP^R^ emerged in Northern Africa in the early 1990s followed by an epidemic spread across Africa, Asia, and Europe [13], coinciding with increased fluoroquinolone use in humans and animals worldwide [15, 16]. Poultry are assumed to be a major reservoir of ST198 and recent surveillance studies indicate the circulation of ST198-CIP^R^ clones in poultry farms in Europe and Asia [17–20]. Human *S*. Kentucky infections in Europe and North America are often associated with travel to Asia or Africa [20–25]. In the EU, approximately half of all human *S*. Kentucky cases are acquired outside of the EU [26]. Little information is available on the characteristics and transmission of *S*. Kentucky isolates in Switzerland.

Although uncommon and not well understood, non-typhoidal *Salmonella* (NTS) can persist in patients over an extended period spanning months or even years [27–29]. In a large study in Israel, prolonged (>30 days) NTS carriage was found in around 2 % of all reported salmonellosis cases, of which 65 % presented with relapsing symptoms [27]. Prolonged colonization is frequently observed despite antibiotic treatment, which can be explained by various factors including antimicrobial resistance or tolerance, insufficient antibiotic concentrations at the colonized niches, or the formation of antibiotic persisters [30]. Antibiotic persisters achieve transient antibiotic tolerance by switching into a non-growing or slow-growing state, resulting in reduced antibiotic uptake or low target activity [30].

This study provides an in-depth analysis of the population structure, resistome, and epidemiological links of 70 *S*. Kentucky isolates that caused human infections in the past decade in Switzerland. We further aimed to investigate genetic determinants underlying varying ciprofloxacin resistance and adaptions of isolates from patients with prolonged colonization. Our results reveal transmission clusters caused by both locally circulating and travel-acquired ST198 clones. Inactivating mutations in *ramR*, an AcrAB-TolC expression regulator, contributed to high-level ciprofloxacin resistance and were frequently detected in persisting strains, providing a strategy for survival in the presence of ciprofloxacin.

## Methods

### Bacterial isolates and antimicrobial susceptibility testing

*S*. Kentucky isolates investigated in this study were obtained between 2010 and 2020 at the Swiss National Reference Centre for Enteropathogenic Bacteria and Listeria (NENT). The NENT receives human clinical *Salmonella* isolates from diagnostic laboratories or medical centres in Switzerland. Antimicrobial susceptibility testing with ciprofloxacin (range 0.002–32 µg ml^−1^) was performed using Etest (bioMérieux). For selected isolates, ciprofloxacin susceptibility testing results were confirmed using broth microdilution according to CLSI guidelines [31] in the presence and absence of efflux pump inhibitor PAβN (final concentration 20 µg ml^−1^). Testing with 15 additional antibiotics (amoxicillin/clavulanic acid [20/10 µg], ampicillin [10 µg], azithromycin [15 µg], cefepime [30 µg], cefotaxime [30 µg], cefazolin [30 µg], chloramphenicol [30 µg], fosfomycin [200 µg], gentamicin [10 µg], kanamycin [30 µg], nitrofurantoin [300 µg], streptomycin [10 µg], sulfamethoxazole-trimethoprim [23.75/1.25 µg], and tetracycline [30 µg]) was performed using the disc diffusion method (BD). Isolates with carbapenemase genes were additionally tested using meropenem, imipenem, and ertapenem Etests (range 0.002–32 µg ml^−1^ each; bioMérieux). Breakpoints for ciprofloxacin (susceptible: ≤0.06 µg ml^−1^; intermediate: 0.12–0.5 µg ml^−1^; resistant: ≥1 µg ml^−1^) and other antibiotics (described in Table S5) were interpreted according to CLSI guidelines [31].

### Whole-genome sequencing

A total of 74 isolates were selected for Illumina whole-genome sequencing. These included 70 isolates from 70 patients chosen from every second year within the study time span, comprising isolates from years 2010 (*n*=6), 2012 (*n*=14), 2014 (*n*=14), 2016 (*n*=17), 2018 (*n*=15), and 2020 (*n*=4). For four patients with a prolonged carriage, second isolates were sequenced (listed in Table S1). Additional long-read sequencing was performed for three ESBL-producing isolates (N12-0931, N12-1542, N16-1393) and two isolates from patients with prolonged *S*. Kentucky colonization (N12-0259, N18-2092).

For short-read sequencing, genomic DNA was extracted using the DNeasy Blood and Tissue Kit (Qiagen). Short-read libraries were prepared using the Nextera DNA Flex Library Preparation Kit (Illumina) and sequenced on the Illumina MiniSeq platform with 2×150 bp paired-end chemistries. Illumina read adapters and low-quality bases were trimmed with TrimGalore 0.6.61 (https://github.com/FelixKrueger/TrimGalore) and draft genomes assembled using SPAdes 3.14.1 [32] implemented in shovill 1.1.03 (https://github.com/tseemann/shovill). For long-read sequencing, genomic DNA was extracted using the MasterPure Complete DNA and RNA Purification Kit (Lucigen). Multiplex libraries were prepared using the SQK-LSK109 ligation sequencing kit with the EXP-NBD114 native barcoding expansion kit (Oxford Nanopore Technologies). Libraries were sequenced on a MinION Mk1B device using the FLO-MIN106 (R9) flow cell (Oxford Nanopore Technologies). Basecalling and demultiplexing were performed with guppy 4.2.2 (Oxford Nanopore Technologies) and adapters were trimmed with Porechop 0.2.4. Hybrid assemblies were generated with Unicycler v.0.4.8 [33] using default settings. The genomic location of antimicrobial resistance genes was confirmed in assemblies generated from long-read data alone using flye 2.8.1 [34] with subsequent polishing using Medaka 1.6.0 (https://github.com/nanoporetech/medaka), and Polypolish v0.5.0 [35]. Assembly quality was assessed using QUAST 5.0.2 [36] and CheckM v1.1.3 [37].

### Genome analyses

Isolates were typed *in silico* using mlst 2.19.0 (https://github.com/tseemann/mlst) and SeqSero2 1.2.1 [38]. Genes were annotated with PGAP 2021-01-11.build5132 [39]. Antimicrobial resistance genes and plasmid replicons were identified using ABRicate 1.0.0 (https://github.com/tseemann/abricate) (90 % coverage, 90 % identity) in combination with the ResFinder [40] and PlasmidFinder databases [41], respectively. Plasmids were compared to those available in the NCBI nucleotide collection using BLASTn [42]. Mutations in QRDRs were identified using AMRFinderPlus [43]. Mutations in loci involved in ciprofloxacin import or efflux (*acrAB*, *acrEF*, *acrZ, ompF*, *tolC*) or its regulation (*acrR*, *acrS, marRAB*, *micF*, *phoQP*, *ramRA*, *rob*, *sidA*, and *soxRS*) were detected using Snippy 4.6.0 (https://github.com/tseemann/snippy) and BLASTn implemented in ABRicate 1.0.0 with respective loci from isolate N12-1542 (CP092006) as references.

Core genome SNPs (cgSNPs) were detected from Illumina read data using the Snippy pipeline 4.6.0 with the chromosome of strain N12-1542 (ST198-CIP^R^ subclade; CP092006) as reference. Phages, IS elements, and repeat regions in the reference chromosome were identified with PHASTER [44], ISEScan 1.7.2.3 [45], and NUCmer 3.1 [46] and masked before read-mapping. Recombinant regions in the Snippy core genome alignment were identified with gubbins 2.4.1 [47]. Maximum-likelihood phylogenetic trees were constructed from the recombination-free cgSNP alignment using IQ-TREE 2.0.3 [48] with the generalized time-reversible (GTR) model with gamma distribution, 100 bootstraps, and the number of invariant sites (-fconst option), which were determined from the Snippy core genome alignment using SNP-sites 2.5.1 (-C option) [49]. cgSNP distances were determined from recombination-free cgSNP alignments with snp-dists 0.7.0 (https://github.com/tseemann/SNP-dists). When mentioned, the CFSAN SNP pipeline 2.2.1 [50] was used as an alternative approach to determine cgSNP distances. Isolates within 10 cgSNPs of each other were considered a potential cluster for further epidemiological investigation.

Publicly available pre-assembled genomes of *S*. Kentucky ST198 isolates from human and non-human sources and associated metadata were obtained from EnteroBase (accessed on 29 November 2021) [51]. Assemblies with N50 <50 kb, >50 000 low-quality bases, or unusual genome sizes (<4.7 or >5.1 MB) were excluded. A phylogeny of global isolates was constructed from an assembly-based core genome alignment generated with parsnp 1.5.3 [52]. Phages, IS elements, repeat regions, and recombinant sites were masked from the alignment, and a maximum-likelihood phylogenetic tree was constructed with IQ-TREE 2.0.3 as described above.

Whole-genome SNPs (wgSNPs) in putative same-strain isolates from patients with prolonged colonization were identified by mapping reads to the draft or, when available, complete assembly of respective first-isolates using the Snippy pipeline 4.6.0. Identified wgSNPs were additionally manually inspected and curated using the variant detection module in CLC Genomics Workbench 21.0.4. Core genome MLST (cgMLST) profiles were called using chewBACCA 2.8.5 [53] with the *S. enterica* scheme from INNUENDO (available at https://zenodo.org/record/1323684#.YfpYCurMKUk), which includes 8558 loci and was curated from the EnteroBase wgMLST scheme [51]. Pairwise distances for alleles present in all investigated genomes were calculated using cgmlsts-dists 0.4.0 (https://github.com/tseemann/cgmlst-dists). Comparisons of genomic regions were generated with EasyFig 2.1 [54].

### Epidemiology

Patient data including age and residential location were retrieved from the NENT database. A map showing the geographic distribution of clinical cases was generated in R 4.0.3 [55] with the sf [56] and ggplot2 [57] packages. Geometric and demographic data was retrieved from the Swiss Federal Statistical Office. Travel-associated cases were defined as those with reported travel within 28 days before symptom onset. The travel history of ST198 salmonellosis patients from England and Switzerland was analysed alongside the phylogeny to determine potential geographical sources of detected clusters. Cases with putative prolonged carriage (i.e. multiple isolates from the same patient obtained >3 weeks apart) were classified as convalescent carriage (3 to 12 weeks), temporary carriage (3 to 12 months), and chronic carriage (>12 months) [58].

## Results

### Prevalence and distribution of serovar Kentucky among human clinical *Salmonella* isolates in Switzerland

**Fig. 1.**
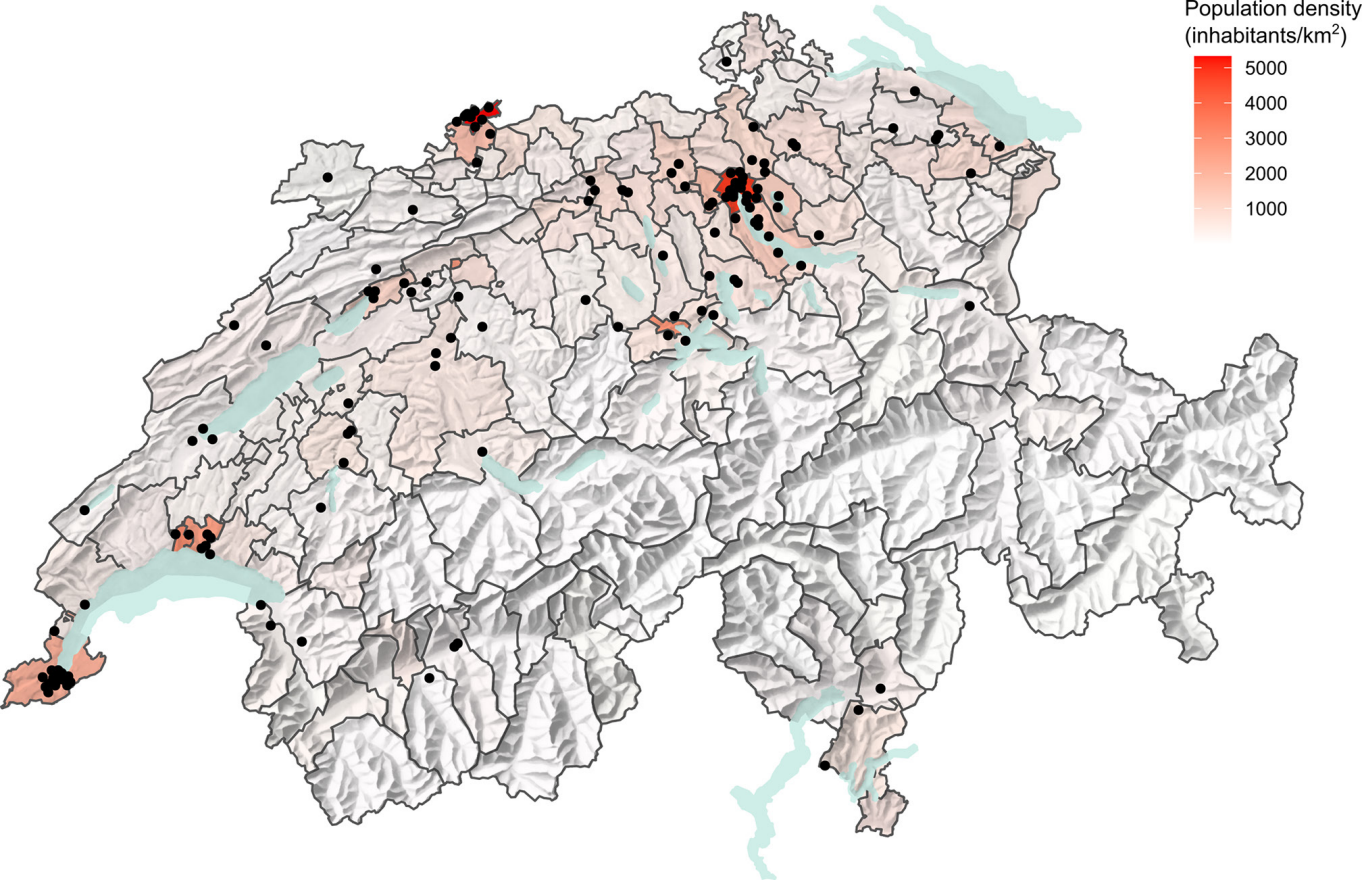
Geographic distribution of human *S*. Kentucky infections in Switzerland. The map shows the geographic location of 135 deduplicated cases (black dots) reported between 2010 and 2020 based on the patients’ postal codes. For 21 cases, the patients’ location was not reported. Overlaying dots were scattered to enable clear visualisation. The population density in each district is shown according to the scale bar with data from 2020.

Between 2010 and 2020, the Swiss National Centre for Enteropathogenic Bacteria and Listeria (NENT) identified a total of 198 *S*. Kentucky isolates (nine to 30 isolates received annually) from 156 patients, accounting for 1.4 % of all received clinical *Salmonella* isolates (*n*=14496). The isolates were recovered from stool (*n*=154), urine (*n*=23), blood (*n*=2), inguinal swabs (*n*=2), or undefined human clinical samples (*n*=17). From 22 patients, multiple (*n*=63) isolates were received. For 16 patients (10.3 %), prolonged carriage of >3 weeks was reported, including nine patients with convalescent, three patients with temporary carriage, and four patients with chronic carriage. *S*. Kentucky cases occurred across all age groups, including infants of 1 to 12 months of age (six patients, 4 %). The densely populated districts of Geneva, Zürich, and Basel (13 % of the Swiss population) together accounted for 30 % of all patients with *S*. Kentucky infections and reported residential location (Fig. 1).

### Population structure and epidemiological links of *S*. Kentucky ST198 isolates from Switzerland

To investigate the diversity of *S*. Kentucky, we analysed whole-genome sequences of 70 isolates obtained between 2010 and 2020 from distinct patients. The selected isolates were recovered from stool (*n*=54), urine (*n*=8), blood (*n*=1), or inguinal swab (*n*=1) samples. For six isolates, the sample type was unknown. All isolates were confirmed as *S*. Kentucky by *in silico* serotyping. Most isolates belonged to ST198 (*n*=64, 91 %; including one isolate with a truncated *purE*); other sequence types included ST314 (*n*=3), ST152 (*n*=1), ST696 (*n*=1), and ST8979 (*n*=1).

**Fig. 2.**
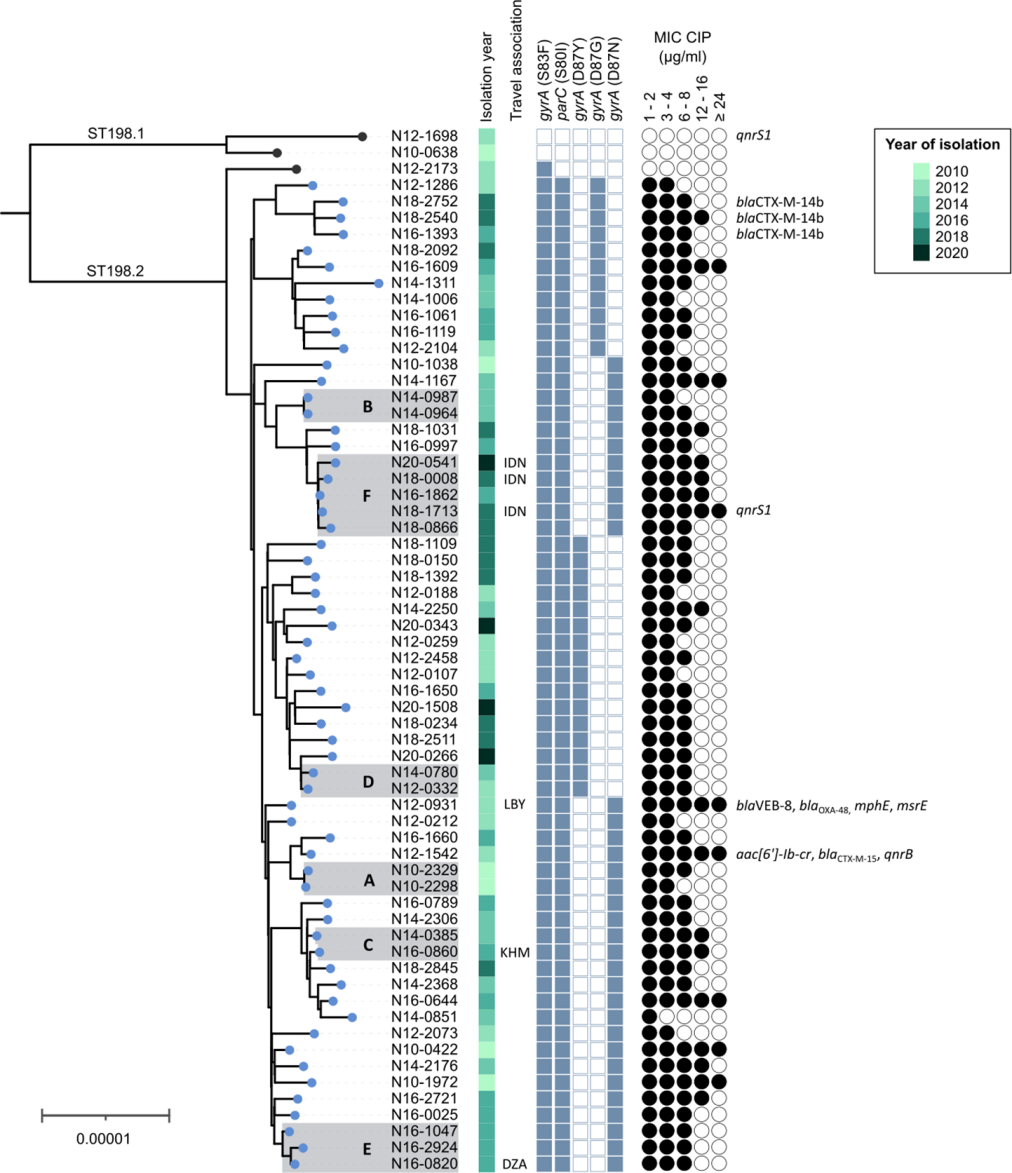
Maximum-likelihood phylogenetic tree of 64 *S*. Kentucky ST198 isolates from Switzerland. Clusters of isolates separated by <10 pairwise cgSNPs are shaded in grey and annotated (A – F). The isolation year is labelled according to the legend. Isolates associated with recent travel are annotated (DZA: Algeria; IDN: Indonesia; KHM: Cambodia; LBY: Libya); for all other isolates, information on the patients’ travel history was unavailable. The presence of mutations in quinolone-resistance determining regions (QRDRs; *gyrA* and *parC*) and minimum inhibitory concentrations (MIC) of ciprofloxacin (CIP) determined using Etest are shown. The presence of acquired genes associated with resistance to azithromycin (*mphE*, *msrE*), carbapenems (*bla*_OXA-48_), ciprofloxacin (*aac(6′)lb-cr*, *qnrB*, *qnrS*), or third-generation cephalosporins (*bla*_CTX-M_, *bla*_VEB-8_) is annotated. Tips of isolates belonging to the CIP^R^ subclade are coloured in blue. The scale bar indicates the number of substitutions per site in a 4.56 Mbp core genome alignment. The tree is based on 921 recombination-free variant sites identified by mapping reads to the chromosome of isolate N12-1542. The tree was visualized in iTOL v5 [83].

For the 64 ST198 isolates, pairwise cgSNP distances and the population structure were determined (Fig. 2). Of 62 isolates belonging to ST198.2, 61 contained three QRDRs mutations in *parC* codon 80 (S80I), *gyrA* codon 83 (S83F), and *gyrA* codon 87 (D87G, D87N, or D87Y) and were thus assigned to the CIP^R^ subclade. Phenotypic ciprofloxacin resistance was shown for all 61 isolates (MICs ranged from 1.5 to >32 µg ml^−1^). Isolate N12-2173, which shared a most recent common ancestor with other ST198.2 isolates, carried only one QRDR mutation (*gyrA* S83F) and was intermediately resistant to ciprofloxacin (MIC=0.125 µg ml^−1^). Two isolates belonged to the ST198.1 subclade and did not carry QRDR mutations. However, one of the two carried the PMQR gene *qnrS1* and was intermediately susceptible to ciprofloxacin (MIC=0.19 µg ml^−1^). The two ST198.1 isolates were collected in 2010 and 2012, while all isolates collected after 2012 belonged to the CIP^R^ subclade (Fig. 2).

Six potential epidemiological clusters with isolates separated by <10 pairwise cgSNPs were detected using the Snippy pipeline. The SNP clusters consisted of two (cluster A, B, C, and D), three (cluster E), and five (cluster F) isolates, respectively, thus comprising 23 % of all investigated ST198 isolates (Fig. 2). The same isolates formed clusters in a cgSNP distance matrix (cutoff <10 cgSNPs) determined using the CFSAN SNP pipeline as an alternative or when applying a distance threshold of ≤5 alleles in a cgMLST analysis among ST198 isolates. An exception was cluster C, which consisted of two isolates differing by seven cgMLST alleles and seven cgSNPs.

For multiple SNP clusters, epidemiological links were supported by a narrow temporal and geographic distribution of the cases (Fig. 2). The bloodstream isolate N10-2326 and stool isolate N10-2329 of cluster A were received within 1 week from different patients residing in the same district and differed by one cgSNP. Similarly, the two isolates within cluster B were received within 1 week from different patients residing in the same region (canton) and differed by two cgSNPs. Isolates from cluster D differed by six cgSNPs and were obtained 2 years apart from neighbouring cantons. The largest cluster (F) consisted of five isolates which differed by one to nine cgSNPs and were obtained over 5 years (2016–2020) from patients of geographically distant regions in Switzerland. Three of the patients had travelled to Indonesia prior to their infection, while for the other two patients travel data was unavailable. Cluster C and cluster E contained one travel-associated case each with patients returning from Cambodia and Algeria, respectively.

### Chromosomal mutations in CIP^R^ subclade isolates associated with varying ciprofloxacin resistance levels

All 61 CIP^R^ subclade isolates harboured three QRDR mutations and were classified as ciprofloxacin-resistant according to CLSI guidelines (minimum inhibitory concentration [MIC] ≥1 µg ml^−1^). Nevertheless, ciprofloxacin resistance levels determined using Etest varied considerably with MICs ranging from 1.5 to >32 µg ml^−1^ (Fig. 2, Table S2). Most isolates (*n*=52) showed medium-level ciprofloxacin resistance (here defined as MIC 3–16 µg ml^−1^); eight isolates showed high-level (≥24 µg ml^−1^), and one isolate low-level (1.5–2 µg ml^−1^) ciprofloxacin resistance. High-level and low-level resistance was confirmed using broth microdilution (Table 1). To investigate potential underlying mechanisms, genes encoding the porin OmpF, the multi-drug efflux pumps AcrAB-TolC and AcrEF-TolC and their regulators were screened for mutations.

**Table 1.** Characteristics of CIP^R^ subclade isolates with high- or low-level ciprofloxacin resistance

Isolate	Ciprofloxacin resistance level (MIC [μg ml^−1^]) ^∗^	Ciprofloxacin +PAβN resistance level (MIC [μg ml^−1^])^∗^	PMQR genes	Mutations in QRDRs	Mutations in genes associated with ciprofloxacin influx/efflux ^2^ (effect)
					
N14-0851	Low [2]	Low [2]	–	*parC* (S80I), *gyrA* (S83F, D87N)	*acrB*: c.2650_2652delGTC (p.V884del)
N10-1972	High [32]	Mid [8]	–	*parC* (S80I), *gyrA* (S83F, D87N)	*ramR*: c.49G>A (p.A17T)
N14-1167	High [32]	Mid [16]	–	*parC* (S80I), *gyrA* (S83F, D87N)	*ramR*: c.101C>T (p.A34V)
					
N10-0422	High [64]	Mid [16]	–	*parC* (S80I), *gyrA* (S83F, D87N)	*ramR*: c.-123A>AG *marR*: c.-46C>A
N12-0931	High [64]	Mid [16]	–	*parC* (S80I), *gyrA* (S83F, D87N)	*ramR*: c.125G>A (p.G42E)
N16-0644	High [64]	Mid [16]	–	*parC* (S80I), *gyrA* (S83F, D87N)	*ramR*: c.531_683dup (p.126_176dup)
N16-1609	High [64]	Mid [8]	–	*parC* (S80I), *gyrA* (S83F, D87G)	*ramR*: c.528_538delTATTGCGCTGG (Y176fs)
N12-1542	High [64]	Mid [16]	*qnrB1*, *aac(6')-lb-cr*	*parC* (S80I), *gyrA* (S83F, D87N)	–
N18-1713	High [32]	Mid [16]	*qnrS1*	*parC* (S80I), *gyrA* (S83F, D87N)	–

*MIC determined by broth microdilution; Etest results are provided in Table S2; and*acrAB, acrEF, acrR, acrS*, *acrZ*, *marRAB*, *micF*, *ompF*, *phoQP*, *ramRA*, *rob*, *sidA*, *tolC*, and *soxRS*

IGR, intergenic region; MIC, minimum inhibitory concentration; PMQR, plasmid-mediated quinolone resistance; QRDRs, quinolone-resistance determining regions

In the low-level resistant isolate N14-0851, an in-frame deletion was detected in *acrB* encoding proton/drug antiporter AcrB (Table 1), plausibly impairing ciprofloxacin export. Of the eight isolates with high-level ciprofloxacin resistance, two harboured PMQRs, and six harboured mutations at the *ramR* locus, which encodes a repressor (RamR) of a transcriptional activator (RamA) of AcrAB-TolC expression (Table 1). One of the six isolates additionally carried a SNP in the promoter region of the *marRAB* operon, another major regulator of AcrAB-TolC expression [10]. Susceptibility testing in the presence of PAβN, an efflux pump inhibitor, decreased MIC values of highly resistant isolates two- to eight-fold but did not show an effect on the low-level resistant isolate N14-0851.

Among the isolates with medium-level ciprofloxacin resistance, one (N12-2073) contained a missense mutation in *acrB* (c.1655>T [p.M552K]); no mutations in the *marRAB* or *ramRA* loci were found (Table S3). The genes *acrEF*, *acrZ*, *ompF*, and *tolC* and the regulatory genes *acrR*, *acrS*, *micF*, *phoQP*, *rob* and *sidA* were unmodified (absence of non-synonymous mutations or indels) in all 61 isolates. Absence (in N14-1311) or IS*26*-mediated disruption (in N18-2092) of the *soxRS* locus (transcriptional regulator) did not impact ciprofloxacin susceptibility (MIC=8 µg ml^−1^ each).

### Acquired antimicrobial resistance genes in ST198 isolates

The two ST198.1 isolates were susceptible or intermediately susceptible to all tested antibiotics (Table S2). By contrast, most of the 61 CIP^R^ subclade isolates were resistant to ampicillin (beta-lactam; 89 %), gentamycin (aminoglycoside; 54 %), streptomycin (aminoglycoside; 57 %), and tetracycline (85 %), in addition to ciprofloxacin (100 %) (Table S2). The most frequent acquired antimicrobial resistance genes among CIP^R^ subclade isolates were *aac(3)-Id* (aminoglycoside acetyltransferase; 57 %), *aadA7* (aminoglycoside nucleotidyltransferase; 69 %), *bla*_TEM-1b_ (beta-lactamase; 82 %), *sul1* (sulfonamide resistant dihydropteroate synthase; 80 %), and *tetA* (tetracycline efflux pump; 85 %) (Table S4). These genes as well as the aminoglycoside phosphotransferase genes *aph(3'')-Ib* (*strA*) and *aph(6)-Id* (*strB*), which were detected in 28 % of the CIP^R^ subclade isolates, have previously been linked to the SGI1-K variant [59, 60]. The chromosomal resistance gene *aac(6')-Iaa* (aminoglycoside acetyltransferase) was present in all isolates, including those that were susceptible to all tested antibiotics. *aac(6')-Iaa* is homologous to *aac(6′)-Iy*, which is cryptic and endogenous to *Salmonella* [61, 62].

Five ST198 isolates were resistant to the third-generation cephalosporin cefotaxime and carried the extended-spectrum beta-lactamase (ESBLs) genes *bla*_CTX-M-14b_ (*n*=3), *bla*_CTX-M-15_ (*n*=1), or *bla*_VEB-8_ (*n*=1; isolate N12-0931) (Fig. 2). N12-0931 additionally carried the macrolide resistance genes *mphE* and *msrE* and showed phenotypic azithromycin resistance.

**Fig. 3.**
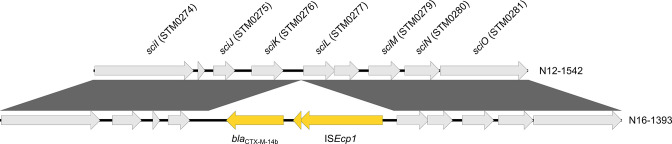
Genetic context of the *bla*_CTX-M-14b_ transposition unit in isolate N16-1393 and the corresponding region of an intact SPI-6 T6SS in the chromosome of isolate N12-1542. Shaded boxes between sequences indicate homologous regions. Genes were identified with PGAP and T6SS genes annotated according to Folkessen *et al*. [84]. The corresponding locus names in *S*. Typhimurium LT2 are given in brackets. The figure was generated with Easyfig 2.1 [54].

For five isolates of the CIP^R^ subclade, chromosomes and plasmids were completely assembled by combining long- and short-read sequencing data. These included three ESBL-producers (N12-0931, N12-1542, N16-1393) and two isolates from patients with prolonged *S*. Kentucky colonization (N12-0259, N18-2092). The isolates harboured six to 14 distinct resistance genes, many of which were co-located in variable combinations in AMR gene clusters. The clusters were typically flanked by IS*26* elements and identified at distinct sites in each isolate: at the chromosomal *trkH*, *soxR*, *rbsK*, or SGI-1 loci or on a large IncHI2/IncHI2A plasmid (Table 2). In addition to its AMR gene cluster, N16-1393 harboured a transposition unit comprising *bla*_CTX-M-14b_ and IS*Ecp1*. This unit was integrated chromosomally between the *sciK* (also termed *hcp1*; STM0276) and *sciL* (also termed *tae4*; STM0277) genes (Fig. 3), which are located on the pathogenicity island SPI-6 and encode components of a type VI secretion system (T6SS), an important virulence factor during *S. enterica* infection [63]. N12-0931 harboured *bla*_OXA-48_ on a 63 499 bp IncL plasmid but was phenotypically susceptible to meropenem, imipenem, and ertapenem (Table S2). *bla*_OXA-48_ is known to spread on highly conserved plasmids [64]. A search of the NCBI nucleotide collection identified five *K*. *pneumoniae* genomes with identical p_OXA-48_ plasmids (63 499 bp length, 100 % query cover and sequence identity; including CP068872.1), which were recovered in the Netherlands. Notably, a stay in Northern Africa was documented in a case report for the patient (P5) before isolation of this highly resistant strain [65].

**Table 2.** Location of antimicrobial resistance genes in complete genome assemblies of five ST198 isolates

Isolate	Antimicrobial resistance genes or gene clusters	Location
N12-0259	*aac (3)-Id, aadA7, qacEΔ1, sul1, tetA*	Chromosomal: into *rbsK* ^∗^
	*bla*_TEM-1b_	Chromosomal: upstream of *yidY* (SGI-1 integration site)
N12-0931	*aac(6')-Ib, armA, bla*_TEM-1b_ *, bla*_VEB-8_ *, qacEΔ1, msrE, mphE, sul1, tetA*	Chromosomal: near *trkH*
	*bla*_OXA-48_	IncL plasmid (63 kp)
N12-1542	*aac (3)-IIa, aac(6')-Ib-cr, ant(3'')-Ia, aph(3'')-Ib, aph(6)-Id, bla*_CTX-M-15_ *, bla*_OXA-1_ *, bla*_TEM-1b_ *, catA1, catB3Δ, dfrA14, qnrB1, sul2, tetA*	IncHI2/IncHI2A plasmid (341 kb)
	*bla*_TEM-1b_	Chromosomal: upstream of *yidY* (SGI-1 integration site)
N16-1393	*aac (3)-Id, aadA7, aph(3')-Ia, aph(3'')-Ib, aph(6)-Id, qacEΔ1, sul1, tetA*	Chromosomal: into SGI-1 ^†^
	*bla*_CTX-M-14b_	Chromosomal: between *sciK* and *sciL*
N18-2092	*aadA2, catA1, dfrA12, qacEΔ1, sul1*	Chromosomal: into *soxR*
	*bla*_TEM-1b_	Chromosomal: downstream of *bsmA*

*two genes were annotated as *rbsK* by PGAP; genes were integrated into *rbsK* located upstream of *fucP*

†according to the assembly generated with flye; in the assembly generated with Unicycler, the resistance gene cluster was located on a 20 kb circular element

### *S*. Kentucky from Switzerland in a global context

**Fig. 4.**
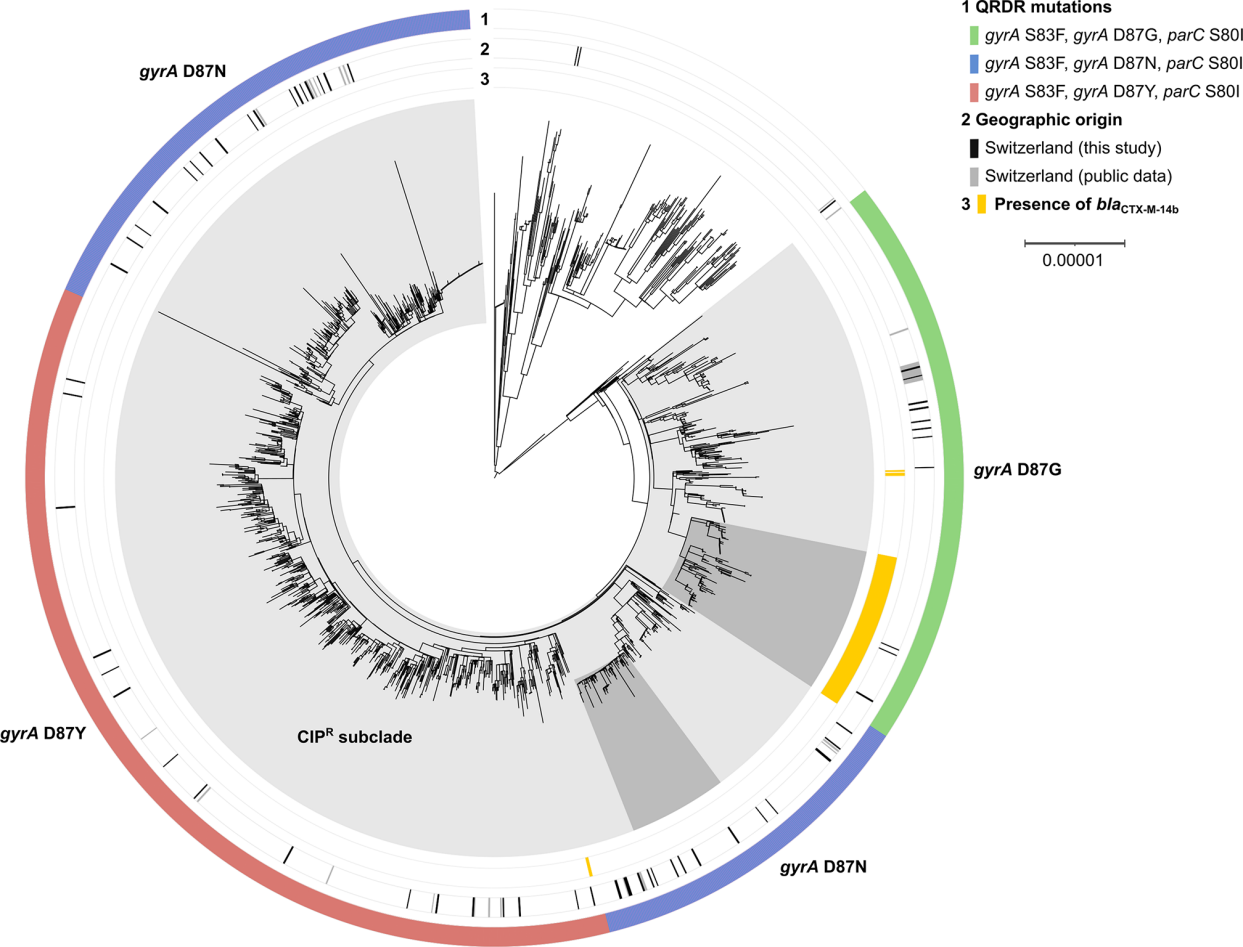
Maximum-likelihood phylogenetic tree of 1853 ST198 isolates. Genomes of 64 ST198 isolates from this study and 1789 publicly available assemblies from EnteroBase were included. The tree is based on 7615 recombination-free polymorphic sites identified in a multi-alignment-derived 3.16 Mbp core genome. Mutations in quinolone-resistance determining regions (QRDRs) (ring 1) and the presence of *bla*_CTX-M-14b_ (ring 3) are indicated. Isolates sequenced as part of this study (black bar, ring 2) and Swiss isolates identified among the public genomes (grey bar, ring 2) are labelled. The CIP^R^ subclade is shaded in light grey. Two branches discussed in the text (a branch characterized by the presence of *bla*_CTX-M-14_ and a branch associated with travel to Indonesia) are shaded in dark grey. The tree was visualized in iTOL [83].

To investigate the distribution of the 61 Swiss *S*. Kentucky ST198 isolates in a global context, pre-assembled genomes from 1789 additional ST198 isolates (listed in Table S6) available on EnteroBase were included in the analysis. The isolates (here termed EnteroBase isolates) originated from 62 countries, with the largest share (47 %) being represented by isolates from the UK (Table S6). A phylogenetic tree was constructed based on cgSNPs identified in an assembly-based multi-alignment (Fig. 4). Consistent with the population structure obtained for the 61 local isolates (Fig. 2), the global isolates with distinct QRDR mutations in *gyrA* clustered separately (Fig. 4). Isolates encoding GyrA D87G (GAC to GGC; *n*=371) and D87Y (GAC to TAC; *n*=664) formed monophyletic branches, respectively. Isolates encoding the GyrA D87N mutation (GAC to AAC; *n*=547) appeared in paraphyletic branches which embedded the D87Y-associated branch.

Within the D87G-associated branch, a monophyletic subbranch consisting of 115 isolates was characterized by the presence of *bla*_CTX-M-14b_ chromosomally integrated downstream of *sciK* (or *hcp1*) (Fig. 4 and Table S7). Isolates within this branch originated from China, the US, and various countries in Europe, and included the three *bla*_CTX-M-14b_-positive isolates (N16-1393, N18-2540, N18-2752) identified in this study (Fig. S1). The emergence and spread of this sublineage in Europe has been reported before [25].

Most Swiss ST198 isolates were distributed across the ST198-CIP^R^ subclade and generally did not show pronounced clustering (Fig. 4). To identify isolates from the EnteroBase collection that genomically matched with isolates sequenced as part of this study, sequencing read data of closely related isolates (<10 pairwise cgSNPs difference to any of the local isolates in a 3.15 Mbp assembly-based core genome alignment of ST198-CIP^R^ subclade isolates) were accessed and cgSNPs determined using two alternative read-mapping based approaches, the Snippy and the CFSAN pipeline.

When applying a strict threshold of <5 pairwise differing cgSNPs to define putative transmission clusters, for 19 of our 61 CIP^R^ subclade isolates at least one and up to 52 matching EnteroBase isolates were identified (Table S8). The Snippy and CFSAN pipelines yielded almost identical results, identifying overall 177 and 187 matching EnteroBase isolates, respectively (Table S9). In some cases, the matching isolates originated from Switzerland but were sequenced as part of other studies. For example, the bloodstream isolate N10-2329 and faecal isolate N10-2298 from the above-described cluster A (Fig. 2) differed by one cgSNP from a meat sample isolate (N10-2202). All three were isolated in 2010 in Switzerland, with the meat isolate recovered 5 weeks before the two clinical isolates. Similarly, isolate N12-0259 (obtained in 2012 patient P4) matched (one cgSNPs) with another isolate (N10-2017) that was recovered in 2010 from animal feed in Switzerland.

**Fig. 5.**
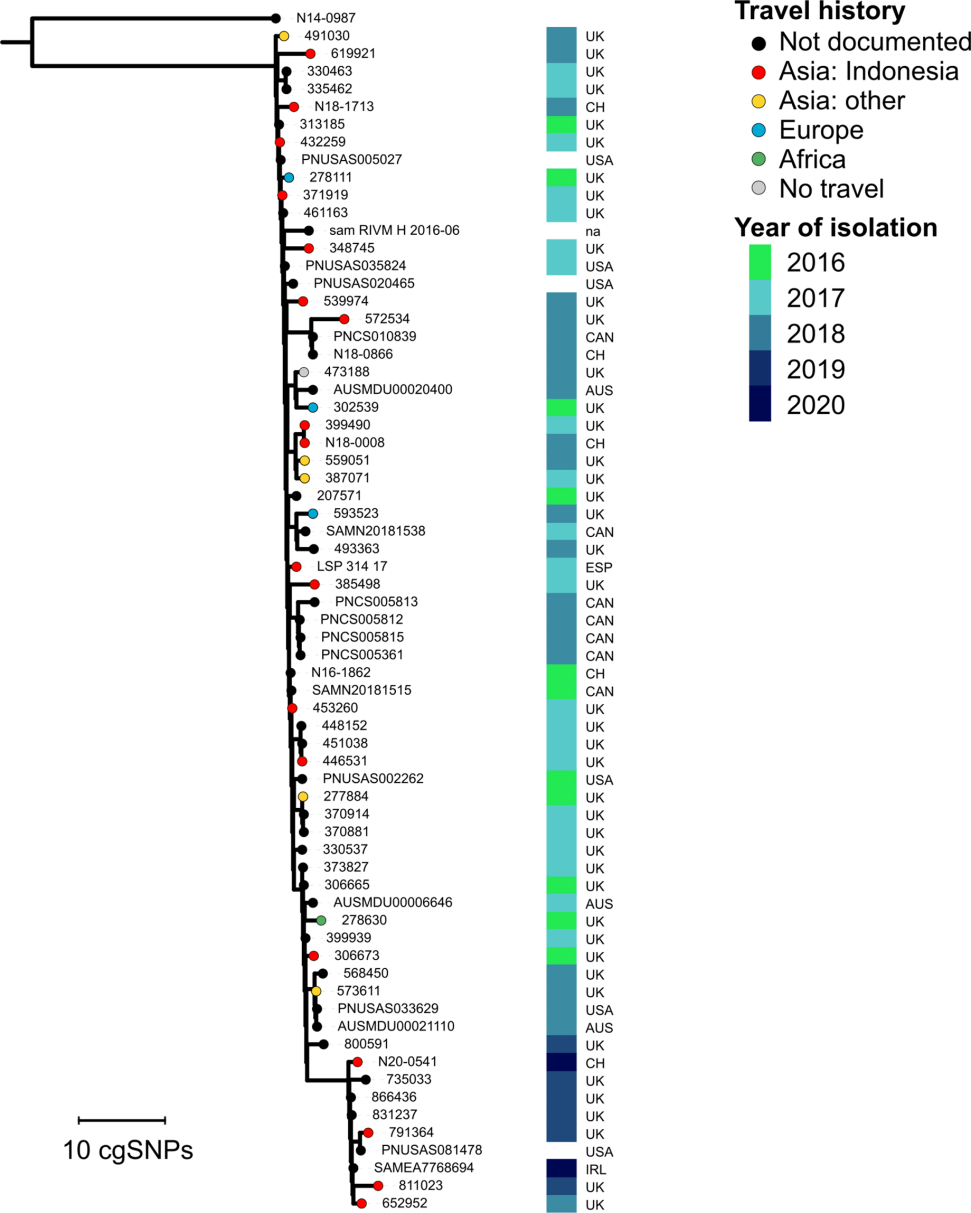
Maximum-likelihood phylogenetic tree of an ST198 SNP cluster associated with travel to Indonesia. Core genome SNP tree for five Swiss isolates from cluster F, 61 matching isolates from EnteroBase, a Spanish isolate (LSP_314_17) previously associated with travel to Bali, Indonesia [23], and N14-0987 as outgroup. Branch tip colours indicate recent travel destinations of the isolates’ carriers according to the legend. The year and country of isolation are labelled. The tree is based on 135 variant sites identified in a 4.8 Mbp core genome alignment generated by mapping reads to the draft assembly N20-0541, which is part of this cluster. The tree was visualized in iTOL v5 [83]. AUS: Australia, CH: Switzerland, CAN: Canada, ESP: Spain, IRL: Ireland, UK: United Kingdom, USA: United States, na: not available.

The largest number of matches was found for the Swiss isolates from the Indonesia-associated cluster F: a total of 61 EnteroBase isolates matched with at least one of the five cluster F isolates (Fig. 5). Pairwise cgSNP distances of the combined 66 isolates ranged from 0 to 16 cgSNPs (mean 4.5) and from 0 to 17 cgSNPs (mean 5.4) when the Snippy and CFSAN pipelines were applied, respectively. When reported, the additional isolates always represented human isolates (*n*=56) collected between 2016 and 2020 in the UK (*n*=43), Canada (*n*=7), the US (*n*=6), or Australia (*n*=3). Of the 43 cases in the UK, 14 cases were linked with travel to Indonesia, five cases with travel to other Asian countries, and four cases with travel in Europe or Africa; one case was not associated with travel within 28 days, and for 19 patients the travel history was not documented (Fig. 5). An additional Spanish isolate (LSP_314_17) reportedly associated with travel to Bali, Indonesia [23] also fell into this cluster (1–10 pairwise cgSNPs) (Fig. 5). Genomic data of this isolate was not available via EnteroBase.

### Genomic changes during prolonged colonization of patients: the *ramR* transcriptional regulator of efflux pumps is a mutational hotspot

For six of the 16 patients with prolonged *S*. Kentucky carriage, we compared the genetic similarity and antimicrobial susceptibility of two or three multi-isolates. The cases included convalescent (*n*=2; 3 to 12 weeks), temporary (*n*=1; 3 to 12 months), and chronic carriage (*n*=3; >12 months), with isolates obtained 1 month to 3 years apart (Table 3). For one patient (P3), an additional stool isolate obtained 1 week after an axilla swab first-isolate was included. Genomic data of the additional same-patient isolates was publicly available (P5 and P6; Table S6) or obtained by additional whole-genome sequencing (P1, P2, P3, and P4; Table S1).

**Table 3.** Mutations, AMR gene acquisition, and phenotypic AMR profiles in ST198 multi-isolates from patients with prolonged colonization

Patient	Isolates	Sampling site	Time after isolation of first-isolate	Genomic changes compared to first-isolate				AMR profile (*gain/loss)		Mutations in *ramRA* or *acrAB* loci vs wild-type variants (effect)
				No. wgSNPs (missense - synonymous - nonsense)	Mutation rate	Other mutations	Gain(+) or loss(-) of ARGs		Ciprofloxacin resistance level (MIC [μg/ml]) ^3^	
**P1**	N18-2511	unknown						AM, CIP, GM, S, TE	Mid (8)	
	N18-2751	stool	1 month	0 SNPs			(+) *fosA3, mphA*	AM, AZM*, CIP, GM, S, TE	Mid (6)	
**P2**	N16-0631	urine						AM, C, CIP, K*, S, SXT, TE	Mid (6)	
	N16-1061	stool	2 months	0 SNPs			(-) *aph(3')-Ia*	AM, C, CIP, S, SXT, TE	Mid (8)	
**P3**	N18-2092	stool						AM*, C*, CIP, S, SXT	Mid (8)	
	N20-2289	urine	25 months	10 SNPs (7 - 2 - 1)	4.8 SNPs/year	two indels	(-) *bla*_TEM-1b_, *catA1*	CIP, S, SXT	Mid (16)	*ramA*: c.23T>C (p.I8T) *ramR*: c.202C>T (p.Q68*)
**P4**	N12-0259	stool						AM, CIP, GM, S, TE	Mid (4)	
	N14-1660	stool	31 months	6 SNPs (5 - 1 - 0)	2.5 SNPs/year	one indel		AM, CIP, GM, S, TE	Mid (6)	
**P5**	N11-2623 ^∗^	axilla						na	na	*ramR*: c.-119_−116delCTCA
	N11-2661 ^∗^	stool	1 week	3 SNPs (3 - 0 - 0)	n.d. ^2^	two indels		na	na	*ramR*: c.125G>A (p.G42E)
	N12-0931	inguinal	5 months	4 SNPs (2 - 1 - 0)	(9.6 SNPs/year)	two indels	(+) *aac(6')-Ib, armA, bla*_OXA-48_ *, bla*_VEB-8_ *, mph(E), msr(E), qacE, sul1*	AM, AMC, AZM, CIP, CTX, CZ, FEP, GM, K, S	High (>32)	*ramR*: c.125G>A (p.G42E)
**P6**	N13-0925 ^∗^	urine						CIP	Low (1.0)	*ramR*: c.528_538delTATTGCGCTGG (p.Y176fs); *acrB*: c.2865dupA (p.R956fs)
	N16-1609	urine	38 months	15 SNPs (7 - 2 - 1)	4.7 SNPs/year	two indels	(+) *aadA2, catA1, dfrA12, sul1*	C*, CIP, SXT*	High (>32)	*ramR*: c.528_538delTATTGCGCTGG (p.Y176fs)

*sequence data retrieved from public depositories; ^2^ SNP rates not determined as isolates were obtained almost concurrently and from distinct sites; ^3^ determined using Etest; MIC determined by broth microdilution for isolates with high- or low-level resistance are listed in Table S2

AM, ampicillin; AMC, amoxicillin/clavulanic acid; AMR, antimicrobial resistance; ARG, antimicrobial resistance gene; AZM, azithromycin; C, chloramphenicol; CIP, ciprofloxacin; CTX, cefotaxime; CZ, cefazolin; FEP, cefepime; GM, gentamicin; IGR, intergenic region; K, kanamycin; MIC, minimum inhibitory concentration; S, streptomycin; SNP, single nucleotide polymorphism; SXT, sulfamethoxazole-trimethoprim; TE, tetracycline fs, frameshift; na, not available;

All multi-isolates belonged to the ST198 CIP^R^ subclade. The second-isolates differed by 0 to 15 wgSNPs from the respective first-isolates, suggesting clonality and persistence in all patients (Table 3). Mutation rates ranged between 2.5 and 9.6 SNPs/year (average 5.4 SNPs/year). However, divergent mutations identified in the three multi-isolates from patient P5, including a unique deletion in the first-isolate, suggested that the three isolates represent distinct subpopulations evolved from a shared common ancestor in P5. This is also supported by an unexpectedly high number of variant sites between the first- and second-isolate, which were collected in the same week, as well as by their recovery from distinct body sites (Table 3). Mutation rates in *Salmonella* were previously estimated to 1–5 substitutions per genome and year [66–68].

Genes controlling the export of ciprofloxacin were identified as a mutational hotspot of isolates from patients with temporary or chronic carriage: out of a total of 39 unique mutations (SNPs/indels), six (15 %) affected the *ramRA* locus or the *acrAB* genes and caused clear changes in the ciprofloxacin MIC (Table 3). These mutations included a deletion, a premature stop codon, and missense mutations in *ramR* and a deletion in the *ramR – ramA* intergenic region. Remarkably, an 11 bp deletion in *ramR* was identified in the first- and second-isolate from patient P6, which were recovered 3 years apart. The first-isolate also contained a frameshifted *acrB* caused by a 1 bp insertion (position 2865), which was no longer found in the second-isolate, indicating the presence of distinct *in situ* evolved subpopulations rather than a reversion of the mutation. Overall, 25 of the 33 detected (deduplicated) SNPs caused amino acid changes in coding regions (*n*=23) or the gain (*n*=1) or loss (*n*=1) of stop codons (Table S10).

Second-isolates from patients P1, P2, P3, P5, and P6 differed in their antimicrobial susceptibility patterns and resistance gene profiles from the respective first-isolates. These included the acquisition or loss of phenotypic resistance to aminoglycoside, azithromycin, beta-lactams, chloramphenicol, and sulfamethoxazole/trimethoprim. All changes could be explained by the acquisition or loss of the underlying resistance genes (Table 3).

## Discussion

Our analysis of 64 ST198 isolates from Switzerland identified both locally and travel-acquired strains as a cause of human disease. Despite a random selection and limited sample size, isolates were often epidemiologically linked as suggested by their narrow temporal and geographic distribution and genetic similarity. Two SNP clusters could be linked with meat or animal feed isolates from Switzerland, pointing towards contamination of the animal food processing chain as a possible source. Poultry and poultry products are assumed to be major vehicles for the spread of *S*. Kentucky ST198 and clonal transmission within poultry farms and processing facilities has been reported, but ST198 has also been identified in other animals and foods [14, 18, 20, 69–71].

The largest cluster of genetically linked ST198 isolates from Swiss patients was associated with recent travel to Indonesia. Screening of public global genomic data of ST198 with a strict threshold of <5 pairwise high-quality cgSNPs identified more than 60 additional isolates that fell into this SNP cluster. The isolates were obtained between 2016 and 2020 in Europe, North America, and Australia, and those with available metadata were predominantly associated with travel to Indonesia (Fig. 5). The cluster might represent a large and prolonged outbreak of a clone that continuously enters the food chain from an unknown contamination source in Indonesia. However, a rapid spread of an endemic clone with various reservoirs and vehicles of infection cannot be excluded. Large and prolonged *Salmonella* outbreaks have been reported before. For instance, an international outbreak of an *S*. Enteritidis clone between 2015 and 2018 in Europe was attributed to the contamination of egg production farms combined with an increased trade of goods [72].

Consistent with previous studies on human *S*. Kentucky in other countries [13, 18, 20, 73, 74], most Swiss *S*. Kentucky isolates belonged to the ST198-CIP^R^ subclade. By contrast, non-human *S*. Kentucky isolates commonly belong to the ST198 ciprofloxacin-susceptible clade ST198.1 or alternative *S. enterica* lineages of the Kentucky serovar, including ST314 and ST152 [11–13]. In phylogenies constructed for local and global ST198 isolates, isolates clustered according to their QRDR mutation in (i) a monophyletic branch characterized by GyrA D87G (GAC to GGC), (ii) a monophyletic branch characterized by D87Y (GAC to TAC), or (iii) a paraphyletic branch characterized by D87N (GAC to AAC). The D87Y-associated branch was embedded in the D87N-associated branch, suggesting that the D87Y-associated lineage may have clonally expanded upon an N87Y (AAC to TAC) mutation. The same arrangement of QRDR mutation-specific branches was observed in earlier studies [14, 25]. Long-read sequencing of five selected isolates identified resistance gene clusters integrated at various chromosomal positions or on plasmids. Only one of those carried a resistance gene cluster in SGI-1, which was identified as a dominant integration site in earlier studies [13, 17, 23].

Approximately 10 % of patients with reported *S*. Kentucky infections were affected by prolonged carriage for at least 3 weeks. All six investigated CIP^R^ ST198 second-isolates from prolonged carriage matched the respective first-isolate, suggesting persistence rather than re-infection with another *S*. Kentucky clone. Persistence may result from their demonstrated multi-drug resistance, rendering antibiotic treatment ineffective. Alternatively, bacteria may survive antibiotic treatment through the formation of biofilms or intracellular persisters, as has been shown for other *Salmonella* serovars [75–77]. In addition to acquisitions and losses of antimicrobial resistance genes, we found evidence of subclonal adaptions among isolates associated with temporary or chronic colonization: despite few SNPs between same-patient isolates, variable mutations at the *ramRA* locus were detected in three out of four investigated cases. RamA transcriptionally activates the drug efflux pump AcrAB-TolC, while RamR represses *ramA* transcription by binding to the *ramR – ramA* intergenic region [10]. In various *Salmonella* serotypes and lineages, including ST198-CIP^R^, inactivating mutations in *ramR* or its binding site were linked to the overexpression of AcrAB-TolC and thus increased AcrAB-TolC production and ciprofloxacin resistance [78–81]. Here, same-patient isolates with apparent reversed deletions or insertions in *ramRA* or *acrAB* suggested that ST198 undergoes divergent adaptions on a subclonal level, possibly upon selective pressure exerted by antibiotic treatment. Remarkably, one identified persistent strain maintained an 11 bp deletion in *ramR* over more than 3 years, possibly in the form of antibiotic persister cells. A frameshifted *acrB* in addition to the deletion in *ramR* found in a urine isolate from this patient may have evolved to restore the metabolic flux: although AcrAB-TolC is the main efflux pump in *Enterobacteriaceae *, its function can be replaced by other efflux pumps [82]. Mutated AcrB was identified in one additional isolate associated with low-level ciprofloxacin resistance. Overall, eight out of ten cases of high- or low-level ciprofloxacin resistance among CIP^R^ subclade isolates could be explained by mutations in *ramR* or *acrB*, and two cases by the acquisition of PMQR genes.

In conclusion, this study demonstrates that the vast majority of *S*. Kentucky infections in Switzerland are caused by ST198-CIP^R^. Reported cases are often epidemiologically linked, with transmission clusters associated with strains that circulate locally or at travel destinations. Prolonged infections were relatively common and frequently associated with mutations in *ramR*, facilitating bacterial survival in the presence of high ciprofloxacin concentrations. Our results demonstrate the value of whole-genome sequencing and global data sharing to trace routes of *Salmonella* contamination and reduce the burden of salmonellosis.

## Supplementary Data

Supplementary material 1

Supplementary material 2
