# Supplementary material for: Epidemiological links and antimicrobial resistance of clinical Salmonella enterica ST198 isolates: a nationwide microbial population genomic study in Switzerland
Source: Microb Genom. 2022 Oct 27;8(10):mgen000877. doi: 10.1099/mgen.0.000877 (PMC9676052; doi:10.1099/mgen.0.000877)
Supplement: Supplementary material 1 [file mgen-8-877-s001.pdf]

Supplementary Material

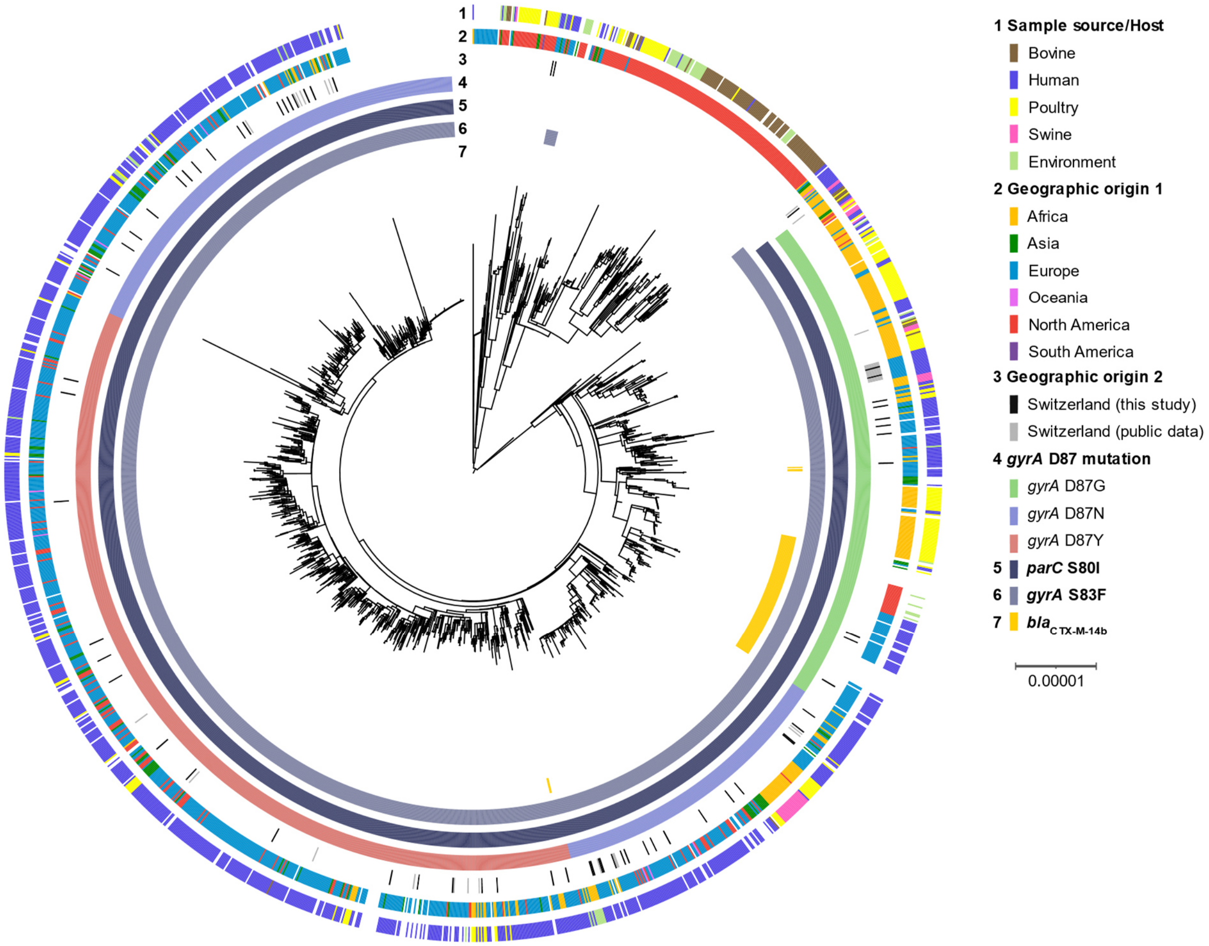

Supplementary Figure 1. Maximum-likelihood phylogenetic tree of 1853 ST198 isolates. Genomes from 64 ST198 isolates from this study and 1789 publicly available assemblies from EnteroBase were included. The tree is based on 7615 polymorphic sites identified in a 3.16 Mbp core genome alignment. The isolates' source (ring 1) and geographic origin (rings 2 and 3) are indicated. Mutations in quinolone-resistance-determining regions (QRDRs) (rings 4, 5, and 6) and the presence of *bla*$_{CTX-14b}$ are labelled (ring 7). The tree was visualized in iTOL.

Supplementary Tables

Supplementary Table S1: Characteristics, genome accession numbers, and assembly metrics of *S.* Kentucky isolates from Switzerland sequenced as part of this study.

Supplementary Table S2: Antimicrobial susceptibility of *S.* Kentucky isolates: minimum inhibitory concentrations (MICs) for ciprofloxacin and disk diffusion inhibition zone diameters (IZD) for 14 additional antibiotics.

Supplementary Table S3: Quinolone resistance determinants and mutations in loci associated with ciprofloxacin influx/efflux among 61 CIP^R subclade isolates.

Supplementary Table S4: Presence of plasmid replicons and antimicrobial resistance genes in *S.* Kentucky isolates from Switzerland.

Supplementary Table S5: Interpretations of antimicrobial susceptibility testing breakpoints according to CLSI guidelines 2020.

Supplementary Table S6: Accession numbers and metadata of 1789 ST198 isolate assemblies from EnteroBase.

Supplementary Table S7: Presence of antimicrobial resistance genes in 1789 ST198 assemblies from EnteroBase.

Supplementary Table S8: Identification of SNP clusters among the Swiss ST198 collection (this study) and ST198 assemblies available on EnteroBase using the Snippy pipeline and Gubbins for removal of recombinant regions.

Supplementary Table S9: Identification of SNP clusters among the Swiss ST198 collection (this study) and ST198 assemblies available on EnteroBase using the CFSAN pipeline.

Supplementary Table S10: Mutations in isolates from patients with prolonged ST198 carriage. Mutations were identified using the Snippy pipeline and manually inspected and curated in CLC Genomics Workbench 21.0.4.
